# Pharmacy Technician Review of Oral Nutritional Supplements (ONS) within Care Homes

**DOI:** 10.3390/pharmacy7010028

**Published:** 2019-03-06

**Authors:** Clare Collins, Catherine Tucker, Carol Walton, Sian Podbur, Steven Barrett

**Affiliations:** 1 Pharmacy Department, Northumbria Healthcare NHS Foundation Trust, Rake Lane, Tyne & Wear, North Shields NE29 8NH, UK; catherine.tucker@nhs.net (C.T.); Carol.Walton2@northumbria-healthcare.nhs.uk (C.W.); steven.barrett@nhct.nhs.uk (S.B.); 2 Dietetics Department, Northumbria Healthcare NHS Foundation Trust, Woodhorn Lane, Ashington NE63 9JJ, UK; sian.podbur@nhs.net

**Keywords:** care homes, pharmacy technicians, waste, deprescribing, nutrition

## Abstract

Up to 42% of patients admitted to care homes are at risk of malnutrition. Oral nutritional supplements (ONS) can be prescribed to increase nutritional intake when diet alone is insufficient to meet daily nutritional requirements. Where ONS are inappropriately initiated or continued beyond treatment goals this can contribute to significant waste and unnecessary costs. This study reviewed whether pharmacy technicians working in care home settings can support the cost-effective use of ONS. A quality improvement project using Plan-Do-Study-Act (PDSA) methodology was undertaken by pharmacy technicians working in care homes to review the prescribing and monitoring of ONS. A sample of 330 residents were reviewed across 5 care homes. 45 residents were prescribed ONS, 16 of whom were unknown to dietitians. In collaboration with the dietetic service an oral nutritional support flow chart was developed and tested. Thirteen of the 16 residents unknown to the dietetic team did not require ONS and could be considered for alternative dietary options. Through collaborative working with dietetic services, pharmacy technicians can support effective use and review of ONS for care home residents, reduce unnecessary prescribing, and ensure appropriate referral to dietitians where indicated.

## 1. Introduction

Oral nutrition support is the provision of dietary advice to patients on how to increase overall nutritional intake and the modification of food and fluid by fortifying food with protein, carbohydrate and/or fat, plus minerals and vitamins. It may include the provision of snacks and/or nutritional supplements as extra nutrition to regular meals and also changing meal patterns. Sip feed is a term which is often used to describe oral nutritional supplements (ONS) that are given to increase nutritional intake [1]. ONS can be a source of nutrients when there is difficulty getting nutrients from food.

It has been estimated that malnutrition (or “undernutrition”) affects over 3 million people in the UK. Of these about 1.3 million are over the age of 65. The British Association for Parenteral and Enteral Nutrition (BAPEN) Nutrition Screening Week surveys (2007-11) have shown that 25–34% of patients admitted to hospital are at risk of malnutrition, 30–42% of patients admitted to care homes are at risk of malnutrition and 18–20% of patients admitted to mental health units are at risk of malnutrition [2]. The best way to detect malnutrition is by the use of malnutrition screening tools, such as the Malnutrition Universal Screening Tool (MUST). This tool assesses patients as being at low, medium or high risk of malnutrition and guides the user to develop individualised care plans for treatment if required and further monitoring [2].

Many studies have reported distinct associations between under nutrition and impaired immune function, increased sepsis, impaired wound healing, impaired muscle function and strength, and increased mortality [3,4]. By giving nutritional supplements to malnourished patients complications such as wound breakdown are reduced by 70% and death by 40% [2]. Care home residents are especially vulnerable, with an estimated 30–42% at risk of malnutrition. One study showed that a group of frail elderly patients given oral supplementation for 12 weeks gained more weight than a group without oral supplementation [5]. Another study showed the benefits of giving ONS at home for 6 months following hospitalization included improved functional status and an increase in independence [6].

Causes of malnutrition include poor dental hygiene, impairment of taste, smell, cognition, attention, manual dexterity and inability to chew or swallow [7]. Explanations for poor dietary intake and malnutrition include unappetising food, absence of dietitian, inadequate nutritional support during intercurrent illness, suboptimal dining environment, dietary restrictions, multiple illnesses, side effects of medicines, and the presence of infections [8,9].

**Figure 1:**
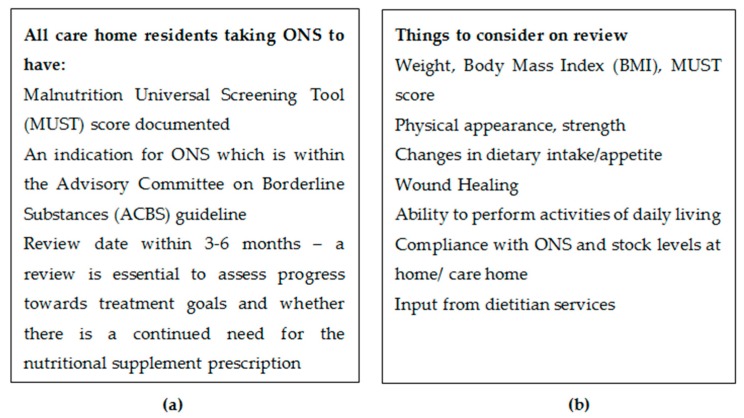
(**a**) Recommended monitoring for residents taking ONS; (**b**) List of considerations during review of ONS [12].

In order to support the nutrition of care home residents, The Department of Health’s National Minimum Standards for care homes for Older People [10] and NICE state that new service users must be weighed on admission to a care home and that their diet and dietary preferences must be assessed. Ongoing nutritional support should be given by keeping a record of nutrition, weight gain or loss, and any appropriate action that has been taken [11,12] (See Figure 1).

A number of studies have highlighted that ONS can help to increase weight gain and nutritional status but often communication and sharing of information is poor [3,7,13]. Different methods to improve compliance have been suggested, including using a multidisciplinary approach (including patients, healthcare workers, and policy makers) or having specific medication rounds for ONS [14,15].

As part of an integrated role working across different sectors of the healthcare system, pharmacy staff from a local acute NHS Trust provided a service to support care home residents across Northumberland, optimising medication and implementing strategies to reduce avoidable medicines waste. Initial observations of the team when reviewing Medication Administration Record (MAR) charts in the care homes included that feeds were often being refused by residents and that there had been examples of excess stock of ONS within the care homes.

As the integrated team were working across both hospital and primary care settings, developing close working relationships with general medical practices was important. The team required access to general practice records and care home administration charts to gain a picture of which ONS were being taken and whether there was any waste. 

A quality improvement project was undertaken within Northumberland care homes to develop a protocol for the effective review and monitoring of ONS. 

## 2. Materials and Methods

### 2.1. Design

The project team consisted of two pharmacy technicians and a dietitian. The model for improvement which incorporates Plan-Do-Study-Act (PDSA) methodology was used to understand the process for reviewing ONS in care homes and to test different solutions. Three PDSA cycles were undertaken over a six months period. Each PDSA stage involved discussion with other members of the project team. Data for ONS prescribing information was collected concurrently while the team also implemented PDSA improvements during the study period. It was clear from the baseline measurement that information would be needed from the different sectors involved: general practice, the care home, hospital discharge letters, and the dietetic service.

### 2.2. Sample

Five care homes were randomly selected from the 70 care homes covered by the pharmacy service. All registered care home residents present in each home would be selected for review. For one month, Pharmacy Technicians reviewed all the MAR charts in the five care homes, as part of the medication review process and any non-compliance with ONS or excess stock of ONS was noted. They discussed ONS with care home nurses/carers who were able to provide information on residents’ current appetite/dietary intake and timing of ONS administration relative to meals. Pharmacy technicians also met with residents and asked how they were managing with their feeds.

In discussion with the care home nurse/carer in each home, the team confirmed whether dietitian services were aware of each individual resident receiving ONS, asked about compliance and residents’ preference for flavour of the ONS and confirmed if there was a planned review date. Where dietician input was required, their role in the review process was to confirm the specialist nutritional plan for residents under their care and to accept referrals for new residents where appropriate.

### 2.3. Quality Improvement Implementation Steps

#### 2.3.1. PDSA Cycle 1

Working closely with each general practice, the team obtained a report on ONS prescriptions for individual residents in the selected homes of the review. The report detailed how often the ONS prescriptions had been issued, the administration instructions and the quantities supplied. The team were then able to compare the prescription information with the resident’s compliance (as noted on the MAR charts) to determine whether there was excess stock. For those residents unknown to the dietetic service, information on which service had initiated the ONS was recorded (see Table 1). The team’s first intervention was to provide feedback to the general practices to update the ONS repeat prescriptions with flavours and quantities to reflect patient preference to reduce excess ONS stock. For any queries with ONS the pharmacy technician would check the hospital discharge letter and contact the prescriber if there was no clear nutritional plan. It was often difficult to find information on hospital discharge letters to confirm whether a referral to the dietitians had been made during the hospital admission and what the ongoing nutritional plan was.

#### 2.3.2. PDSA Cycle 2

The second intervention was to speak directly to the community dietician team to explore the queries and missing information further. It was difficult for the team to find the current and previously documented MUST scores in either the care home notes or the primary care record. Where the ONS was initiated by the general practitioner (GP) there was no standard initiation/review form which contained the nutritional plan and goals. The pharmacy team met with the community oral nutrition lead from the acute NHS Trust to discuss the data and develop an improved model to enable pharmacy technicians to support ONS review. It became clear that some care home residents were unknown to the dietetic service (see Table 2). It was decided for all queries about ONS for care home residents, the pharmacy team would make a direct referral to the community dietetic team via email.

#### 2.3.3. PDSA Cycle 3

The third intervention was made following a review of dietician referrals. In accordance with the NHS Trust’s Information Governance policy, pharmacy technicians within the care home service were provided access to the dietetics database which allowed them to identify care home residents that were unknown to the dietetics service and were being prescribed ONS without dietetics review. The team was also able to verify whether changes to ONS prescriptions that were recommended, following the dietitian review, had been updated on the resident’s records.

**Figure 2:**
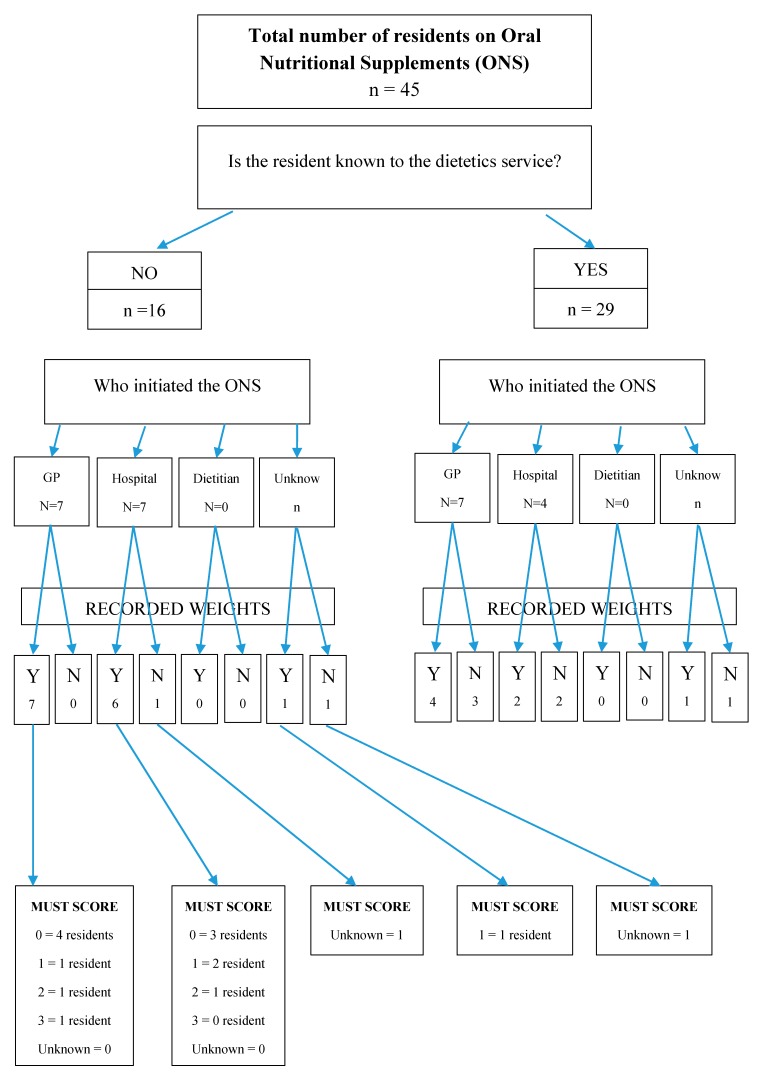
Figure to demonstrate recording of weight and MUST score of residents receiving ONS.

**Figure 3:**
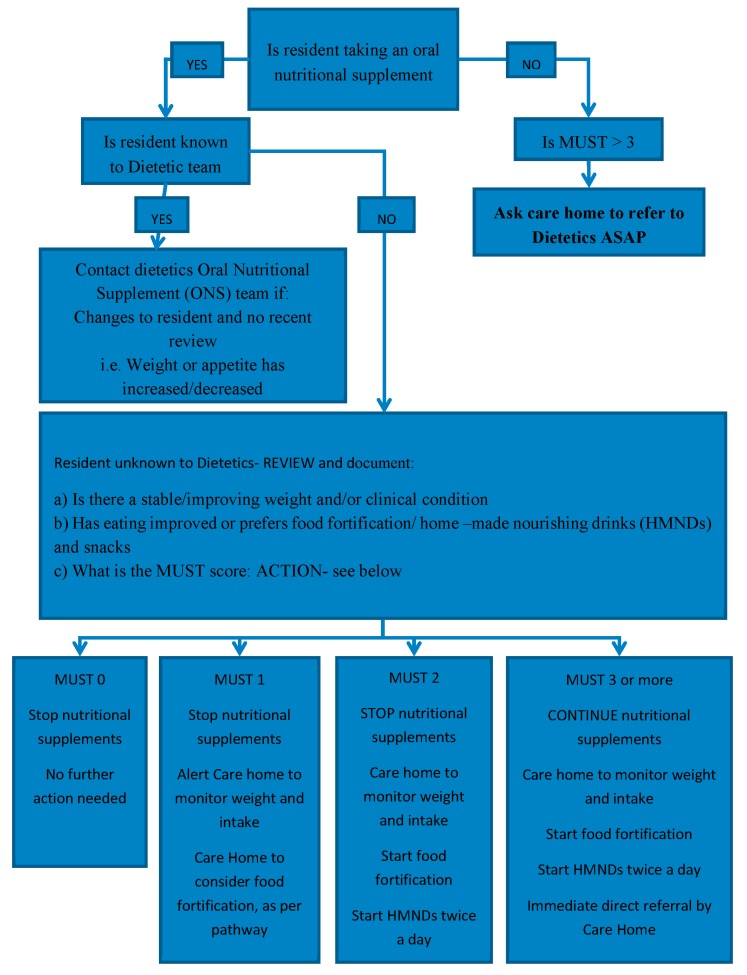
Pharmacy medication review team: oral nutritional support review chart.

The pharmacy technicians were able to calculate the MUST score for those residents unknown to the dietetic service using a web-based calculator [2] (see Figure 2). Through further discussion about residents who were unknown to the dietetic service, a flowchart was developed to help determine the appropriate management according to MUST score (Figure 3). The flow chart included guidance on which residents require a review by a dietitian.

### 2.4. Ethics

This quality improvement project was discussed with our internal research team and NHS ethics was deemed unnecessary.

## 3. Results

Pharmacy technicians reviewed five care homes with a total of 330 residents. Of these, 45 residents were prescribed oral feeds and 16 of those residents were unknown to the dietetic team.

Using the Pharmacy Oral Nutritional Support review flow chart, it was confirmed that 13 of the 16 residents unknown to the dietetic team did not require ONS but could be considered for alternative options according to the MUST score (see Figure 3). One resident had a MUST score of 3 and so was referred to dietetic services for further follow up and two residents had no weights recorded and so they needed further monitoring and subsequent follow up.

Overall, there were 23 of the 45 residents whose ONS were initiated by either the GP or hospital. There were six residents for whom it was not clear which service had initiated the ONS.

## 4. Discussion

In the five care homes visited, the team found 16 residents who were unknown to the dietetics service. The flow chart enabled these residents to be effectively reviewed and appropriate management initiated. There were a number of examples where residents were taking ONS less than the prescribed frequency which had led to a build-up of stock. Where such non-adherence occurred, in most cases there was no clear documentation of this or adjustment to the ONS prescriptions and/or MAR charts to avoid further build-up of ONS stock. By having access to the GP practice records and following discussion with the dietitian/GP/resident, the pharmacy technicians were able to update the quantities of ONS for future acute or repeat prescriptions and thus reduce the excess supply of ONS for residents who were not consistently taking the ONS as prescribed. It also provided information about when the residents had been reviewed and allowed the technicians to contact the most appropriate person where changes were needed.

One of the reasons for non-compliance with ONS was the palatability and the resident’s preference for different flavours. Therefore, it is important to incorporate the resident’s choice when prescribing ONS. Within the pharmacy technician service, discussion with residents is now prioritised and the preferred flavour is consistently recorded on the prescription(s) within the GP records as part of the review. When initiating ONS for a care home resident, “mixed flavours” should be requested only until taste preferences have been established [16].

If there is variation with compliance of the oral feeds it is important to have this reviewed by the prescriber. Checks should be made to see if it is still appropriate to continue the feed or perhaps consider different formulations or flavours. NICE have issued guidance on nutritional support for adults which suggests that health professionals should review the indication, route, risks, benefits and goals of nutritional support at regular intervals. People having oral nutritional support in the community should be monitored every 3–6 months or more frequently if there is a change in their clinical condition [15]. Decisions about ONS need to be discussed with the patient and their carers to ensure they are fully informed about their treatment and have an opportunity to discuss their nutrition, agree a management plan and have an opportunity to ask questions [17].

Through collaborative discussions this process has enabled joint working with the care home pharmacy technicians, the dietetics team, general practice, and care home staff. This improved sharing of information and communication has led to more cost-effective prescribing through deprescribing of inappropriate ONS, although the study did not measure the overall prescribing costs including the potential additional costs resulting from new dietitian referrals.

Using the developed flow chart has improved the consistency of ONS reviews for residents and ensured appropriate referral to dietitian services where indicated. Pharmacy technicians completing regular medication reviews in care homes are ideally placed to highlight appropriate residents for referral or review and eliminate unnecessary prescribing and administration of these high cost products. The developed method for reviewing the ONS has become part of the routine MAR chart and medication review for the care home pharmacy service.

During this review it was sometimes difficult to find the required information to effectively review the ONS, partly because there was no universal form or template used across the different care settings. ONS are not always initiated by the dietetic service or hospital and within the sample there was also inconsistency in recording of ONS initiation on GP records. Clear communication is important, as well as documentation of specific monitoring data to ensure the nutrition goals are monitored and met.

Some of the residents reviewed had ONS started in hospital which had not been reviewed following discharge. Further research could be performed utilising similar quality improvement study methodology to improve the communication of nutritional plans across organisational boundaries following discharge from hospital.

## 5. Conclusions

Nutrition plays an important part in health and wellbeing. There are many ways to increase overall nutritional intake, including using ONS where appropriate. However, a successful outcome for a nutrition review relies on good communication and documentation of nutritional goals, regular review, and most importantly including patients in the discussion to understand and acknowledge their preferences. Within this study, the practical benefits for multidisciplinary review of ONS include improved quality and efficiency of information gathering and joined up communication, ensuring clinical recommendations are carried out in a timely manner. This study highlights the benefits for pharmacy care home service co-development with inter-professional collaboration and integration across care settings. Further research would be needed to determine whether this type of collaboration is replicable with other professional groups, transferable to other healthcare systems and deliverable on a wider scale.

## Figures and Tables

**Table 1:** Service initiating the oral nutritional supplement.

Care Home	Number of Residents on ONS	Number of Residents on ONS Unknown to Dietetics	Which Service Initiated ONS			
			GP	Hospital	Dietitian	Unknown
1	11	7	4	1	4	2
2	7	1	1	1	5	0
3	9	2	0	2	4	3
4	8	0	6	2	0	0
5	10	6	1	5	3	1
Total	45	16	12	11	16	6

**Table 2:** The number of residents prescribed oral nutritional supplements (ONS) and unknown to the dietetic service.

Number of Care Homes Visited	Total Number of Residents	Residents on ONS	Residents Unknown to the ONS Team	No. of Residents Where ONS Was Stopped Post Review	Annual Calculated Cost of Stopped ONS Post Review
5	330	45 (14% of residents)	16 (5% of residents)	13 (3.9% of residents)	£27,765
